# Extrafollicular Dermal Melanocyte Stem Cells and Melanoma

**DOI:** 10.1155/2012/407079

**Published:** 2012-05-10

**Authors:** James D. Hoerter, Patrick Bradley, Alexandria Casillas, Danielle Chambers, Carli Denholm, Kimberly Johnson, Brandon Weiswasser

**Affiliations:** Department of Biological Sciences, Ferris State University, Big Rapids, MI 49307, USA

## Abstract

Recent studies suggest that extrafollicular dermal melanocyte stem cells (MSCs) persist after birth in the superficial nerve sheath of peripheral nerves and give rise to migratory melanocyte precursors when replacements for epidermal melanocytes are needed on the basal epidermal layer of the skin. If a damaged MSC or melanocyte precursor can be shown to be the primary origin of melanoma, targeted identification and eradication of it by antibody-based therapies will be the best method to treat melanoma and a very effective way to prevent its recurrence. Transcription factors and signaling pathways involved in MSC self-renewal, expansion and differentiation are reviewed. A model is presented to show how the detrimental effects of long-term UVA/UVB radiation on DNA and repair mechanisms in MSCs convert them to melanoma stem cells. Zebrafish have many advantages for investigating the role of MSCs in the development of melanoma. The signaling pathways regulating the development of MSCs in zebrafish are very similar to those found in humans and mice. The ability to easily manipulate the MSC population makes zebrafish an excellent model for studying how damage to MSCs may lead to melanoma.

## 1. Introduction

Cutaneous malignant melanoma is the most serious form of skin cancer with the highest mortality rate. The rate of incidence for melanoma has been rising around the world with the USA reporting about 60,000 cases and 8,000 deaths every year [1]. It is a complex disease with multifaceted etiology that involves both genetic and environmental factors. Most melanomas arise *de novo,* but some are known to arise in preexisting nevi or moles [2]. Melanomagenesis is often medically described as a gradual transformation of a cutaneous melanocyte in the basal layer of the epidermis, enabling the stepwise metamorphosis from nevus to radial-growth phase, to vertical growth, and finally to metastatic malignant melanoma [3].

The identity of the original target cell that acquires the requisite DNA lesions for transformation to melanoma still remains elusive. The traditional hypothesis is that cutaneous melanocytes progressively accumulate mutations in oncogenes and tumor suppressor genes over a long period of time during exposure to UV, leading to uncontrolled proliferation, acquisition of invasive properties, and ability to metastasize [4, 5]. An alternative hypothesis is that melanoma begins in an extrafollicular dermal melanocte stem cell (MSC). Although MSCs have not been definitively isolated, they are likely to exist based on the presence of stem cell markers on putative melanocyte precursors in the dermis [6] and on multipotent stem cells in the dermis that have been isolated and shown to differentiate into melanocytes in human skin constructs [7–9]. Understanding the mechanisms controlling self-renewal, expansion, and differentiation of the extrafollicular MSCs will provide greater insight into the possible mechanisms for the neoplastic development of malignant melanoma. UV-induced mutation may alter the normally tightly controlled process of self-renewal, expansion, and differentiation of MSCs as well as their exit from the stem cell compartment. Different forms of melanoma may reflect the stage in the melanocytic differentiation pathway where transformation occurs.

 The purpose of this review is to summarize the data supporting the existence of extrafollicular dermal MSCs and to explain how the neoplastic development of malignant melanoma is better understood when it is viewed as having its earliest origins in an extrafollicular MSC or in a melanocyte precursor derived from it, rather than in a mature, fully differentiated cutaneous melanocyte in the basal layer of the epidermis [9–11].

## 2. Results and Discussion

### 2.1. Epidermal Melanocytes

In humans melanocytes are melanin-producing cells that comprise only about 5–10% of all of the body's skin cells. Each melanocyte is located on the basal layer of the epidermis and immediately surrounded by approximately five keratinocytes. Through multiple dendritic extensions, melanocytes establish contact with an additional 35–40 keratinocytes to form an epidermal melanin unit. The epidermal melanin unit is thought to be a symbiotic relationship between the melanocyte and the pool of associated keratinocytes. The melanocyte maintains this homeostatic ratio throughout its lifespan [12, 13]. When a keratinocyte is exposed to sunlight, it relays signals to its connecting melanocyte and turns on the pathways leading to melanin synthesis and the formation of melanosomes. The melanosomes are then transferred from the melanocytes to the keratinocytes through the dendritic connections, collectively forming a temporary microparasole over the skin. Melanin serves to absorb UV photons and to quench highly reactive oxygen radicals, providing the skin with a first line of defense from the damaging rays of UV radiation [14].

Although melanocyte mitosis has been rarely observed *in vivo* under normal physiological conditions, this is not to suggest that melanocytes have lost their ability to divide. A small percentage of melanocytes appear to divide during normal skin homeostasis [15]. When melanocytes are separated from their normal physiological niche and cultured in isolation, they divide continuously with doubling times of 48–96 hrs [16, 17]. The precise mechanisms that control the organization and the number of melanocytes in the epidermis are unknown. Several studies strongly suggest that keratinocytes interact with melanocytes via growth factors, cell surface molecules, or other factors related to proliferation and differentiation of the epidermis [18–20]. Continuous crosstalk between keratinocytes and melanocytes is required to maintain a ratio of approximately 5 : 1 of keratinocytes to melanocyte on the basal epidermal layer [21, 22]. When melanocytes become transformed, they escape the controlling influences of the surrounding keratinocytes and develop new cellular connections with fibroblasts and endothelial cells to support their growth and invasion [23].

### 2.2. Evidence for Extrafollicular Dermal MSCs

Even though extrafollicular dermal MSCs have not yet been isolated in human skin, it is plausible that they do exist based on indirect evidence of stem cell markers, tissue culture studies, and repigmentation patterns observed in patients with vitiligo. Although it is always possible that existing melanocytes could be stimulated to divide and to replace damaged melanocytes, given the vital role that melanocytes play in constantly protecting the skin against UV radiation, the skin is likely to have a pool of MSCs in a well-protected area of the dermis to draw upon to replace any damaged melanocytes in the basal layer of the epidermis. The signal to replace a melanocyte may originate from the surrounding keratinocytes that comprise the epidermal melanin unit. Fully differentiated melanocytes, like other cells of the skin, are constantly exposed to solar UV. After a sufficient number of lesions, a melanocyte that is damaged beyond the capability to repair itself is likely to be eliminated via an apoptotic pathway. Following apoptosis the homeostatic balance between the melanocyte and surrounding keratinocytes will be disrupted thus signaling a melanocyte stem cell in the dermis to differentiate and migrate to the epidermis to reestablish the melanocyte : keratinocyte ratio. For example, this may occur after a severe sunburn or during body growth from childhood to adult stature to accommodate increased skin surface area.

It was suggested many years ago that extrafollicular dermal melanocytes may be derived from pluripotent cells that migrate from the neural crest to the skin via the paraspinal ganglia and their peripheral nerves [24–26]. Here they give rise to melanocyte precursors when replacements for epidermal melanocytes are needed in the postnatal skin [27]. Many studies support the hypothesis that peripheral nerves may function as a MSC niche from which cutaneous melanocytes are recruited during skin regeneration and repair [28–32]. The most convincing evidence comes from the isolation of human multipotent stem cells from the dermis of glabrous skin that are capable of self-renewing and expressing the neural crest stem cell markers NGFRp75 and nestin. When these stem cells are placed in human skin reconstructs, they migrate to the basal epidermis, establish communication with keratinocytes, and differentiate into melanocytes [7, 9]. Other supporting evidence for extrafollicular MSCs in the dermis comes from the observation that Schwann cells, the principal glia of peripheral nerves, display a highly unstable phenotype that can be reversed or induced to transdifferentiate while in culture, indicating that these cells, like stem cells, are permissive for phenotypic changes [33].

The presence of MSC markers KIT and BCL-2 in the basal layer of human epithelia suggests the presence of melanocyte precursors [6]. Another possible marker of a MSC, human CD133 antigen, has been found in stem cell niches comprising the basal layer of human neonatal epidermis [34]. Other evidence for dermal MSC comes from clinical studies in patients with a skin disorder, vitiligo. Vitiligo is an acquired disorder of pigmentation, characterized by depigmented, cutaneous macules resulting from the loss of functioning melanocytes. When a patient with vitiligo was administered an oral dose of 8-methoxypsoralen in combination with UVA therapy to stimulate repigmentation, tyrosinase-positive melanocytes were detected along the basement membrane in the previously depigmented palms. This provides clinical evidence supporting the existence of a MSC reservoir in glabrous skin [35, 36].

The evidence for the presence of extrafollicular dermal MSCs in association with the peripheral nerves is derived from a variety of vertebrates including quail, chicken and zebrafish, suggesting that the developmental pathways regulating MSCs are conserved and that the use of these animal models will likely yield important insights into the origin, regulation and control of MSCs in humans (Table 1).

The presence of stem cell markers which are indicative of cells in the melanocyte lineage, the isolation of multipotent stem cells from the human dermis capable of differentiating into melanocytes, and the capability of melanocytes to regenerate provide substantial evidence that the vertebrate dermis contains an extrafollicular reservoir of MSCs.

There is some evidence to suggest that the bulge region of the hair follicle might serve as a supplemental reservoir of MSCs to replenish melanocytes in the basal epidermal layer of the skin [42]. This study is supported by the finding that MSCs in the bulge region of the murine hair follicle are capable of producing transient amplifying cells with the potential to migrate into empty niches including the skin [43].

### 2.3. Regulation of Extrafollicular Dermal MSCs

The microphthalmia-associated transcription factor (MITF) is the master regulator of melanocyte differentiation, development and survival [44]. It plays a central role in the complex network of interacting genes regulating the migration, survival and proliferation of melanocytes [45–47]. Because of its crucial importance in regulating the development of melanocytes, it is not surprising to find that at different stages of melanocyte development, MITF expression is regulated by an array of cooperating transcription factors that all influence how the MITF promoter responds to developmental signals [45]. MITF is also required to establish the MSC in the follicular niche [48, 49] and for this reason, it is thought to play a role in regulating extrafollicular dermal MSCs.

Two important transcription factors, PAX3 and SOX10, are involved in the regulation of the MITF promoter and thus may also play critical roles in regulating the extrafollicular MSCs [50–52]. Synergistically with SOX10, PAX3 strongly activates MITF expression. These two transcription factors physically interact and bind directly to the MITF promoter [53, 54]. PAX3 is a member of a highly conserved family of transcription factors essential during the early development of many different tissue types and to the preservation the stem cell state. It affects melanocyte proliferation, resistance to apoptosis, migration, lineage specificity and differentiation [55, 56]. This transcription factor functions as a nodal point for coactivators and inhibitory proteins involved in regulating the MSC, as well as its differentiation into a mature melanocyte. Although the PAX3 protein is required for melanocyte development during embryogenesis, it is not found in normal mature melanocytes. PAX3 expression preserves pluripotency and its repression induces differentiation [57].

Wnt and Notch signaling pathways also play key roles in regulating MSCs. WNT signaling regulates quiescence, expansion and differentiation of MSCs by modulating the levels of PAX3, SOX10 and MITF [56–58]. NOTCH signaling pathways are part of an evolutionary conserved complex of signaling pathways and are essential for the maintenance of the immature status of MSCs. In the hair follicles of mouse skin, NOTCH signaling controls the spatial distribution of MSCs and the timing of their differentiation into melanocytes [59, 60]. These signaling pathways may be equally important in regulating both quiescent (out of cell cycle and in a lower metabolic state) and active (in cell cycle and not able to retain DNA labels) MSC subpopulations that may coexist in the dermis in separate yet adjoining locations [61].

Genes regulating the development of MSCs are highly conserved in humans, zebrafish and mice, providing confidence that experimental findings involving MSCs in zebrafish can be translated to humans for understanding the origins of melanoma (Table 2).

### 2.4. Melanoma Begins in an Extrafollicular Dermal MSC

Solar ultraviolet (UV) radiation is the prominent environmental physical carcinogen involved in melanoma. Decades of epidemiologic studies link solar UV radiation to the development of malignant melanoma [80]. Solar UV reaching Earth's surface is a continuum of electromagnetic radiation and is divided into UVA (320–400 nm) and UVB (290–320 nm) wavelengths for purposes of describing the biological effects associated with long- and short-wave UV radiation. The specific contribution of UVA and UVB in the etiology of melanoma is controversial. However, there is adequate evidence to suggest that both UVA and UVB radiation act together and sometimes synergistically to promote the development and progression of malignant melanoma [81]. UVB radiation is less penetrating and is directly absorbed by DNA, causing several mutagenic DNA lesions; UVA radiation on the other hand, penetrates deeper in the skin and indirectly produces its effects by the generation of reactive oxygen species (ROS), leading to oxidative damage of DNA and protein [82]. That fact that ROS is implicated in all stages of multisteps carcinogenesis suggests that UVA may play an important role in the development of melanoma [83]. However, the relative roles of UVA and UVB radiation in the development of melanoma are far from being resolved. One of the most important factors in the cellular ROS defense machinery is the transcription factor Nrf2. It induces the production of a variety of ROS detoxifying enzymes and antioxidants such as glutathione and plays an important role in the protection of the skin against UVA-induced apoptosis [84]. Nrf2-mediated ROS cytoprotection is also thought to be partially responsible for decreased UVB-induced apoptosis in the skin [85, 86]. We speculate that this increased level of protection against apoptosis via Nrf2 may be a double-edged sword. By decreasing apoptosis of damaged cells after exposure to intense and intermittent UV radiation of tanning beds, it may well promote the retention and accumulation of UV-induced damage in quiescent and active MSCs in the dermis.

**Figure 1:**
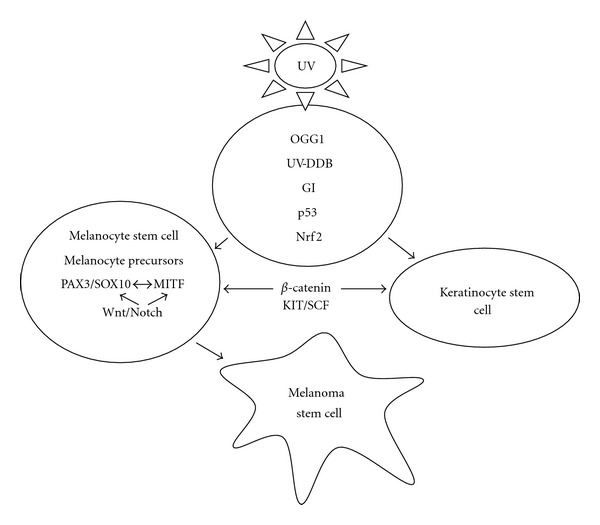
A model summarizing how important signalling pathways, and some DNA repair and transcription factors in melanocyte and keratinocyte stem cells or in their derivatives, might be impaired by UV irradiation, leading to the development of melanoma stem cells. Lower levels of the repair enzyme, human 8-oxoguanine-DNA glycosylase (OGGI), UV-damaged DNA-binding protein (UV-DDB), along with an attenuated p53 apoptotic response will increase survival of cells in the melanocyte lineage with mutational loads and genetic instability (GI). Increased expression of Nrf2 will further prevent UVA-induced apoptosis and thus promote survival of cells, increasing the retention and accumulation of mutations. Mutations in critical genes for transcription factors regulating melanocyte stem cell proliferation and differentiation (MITF, PAX3 and SOX10) or in signaling pathways (Notch and Wnt) will have profound and cascading effects on those pathways regulating the quiescence, expansion and differentiation of melanocyte stem cells. Increased *β*-catenin stimulate proliferation, abnormal differentiation, and self-renewal of melanocyte stem cells. Alterations in c-kit tyrosine kinase receptor (KIT) and its ligand, (stem cell factor (SCF)), will alter the homeostatic balance between keratinocytes and melanocytes. All of these factors may interact and contribute to the transformation of an epidermal MSC into a melanoma stem cell.

Our proposed model (Figure 1) for the origin of malignant melanoma is based on the hypothesis that cutaneous melanoma has its earliest origins in an extrafollicular MSC residing in the dermis of the skin. Under normal environmental conditions, cellular DNA in the MSC will be frequently exposed to various doses and fluence rates of UVA and UVB radiation from sunlight, and in some cases, from exposure to high fluences delivered by tanning beds. UVA radiation because of its longer wavelength will penetrate deeper into the dermal layer of the skin than the shorter UVB wavelengths [87]. Therefore, our model places greater emphasis on the effects of UVA radiation on dermal MSCs in the beginning stages of melanoma. UVA has the potential to inflict DNA damage to the MSC residing in the superficial nerve sheath of peripheral nerves. This scenario is similar to what is found in squamous cell carcinomas where UVA fingerprint mutations are most abundant in the basal germinative layer, suggesting that UVA- rather than UVB-induced DNA damage is an important carcinogen in the stem cell compartment of the skin [88]. Damage to DNA and repair proteins in MSCs will occur via the production ROS, hydrogen peroxide, and superoxide anion [89]. ROS may also give rise to the very reactive hydroxyl radical via Fenton reactions when ferritin, which is known to restrict the availability of iron after UVA irradiation, is impaired or fails to be induced [90, 91]. Short-term exposure to high fluences of UVA in tanning beds provokes an immediate increase in intracellular labile iron before defense mechanisms have time to remove the iron. This provides an ideal environment for generating oxidative reactions leading to increased damage to DNA and proteins [92]. An important repair enzyme, human 8-oxoguanine-DNA glycosylase (OGG1), known to be involved in repairing UVA-induced oxidative DNA damage, is much lower in skin cells of the basal layer of the human epidermis including melanocytes [93]. This suggests that oxidative DNA mutations may also be less efficiently repaired in MSCs. A study showing that epidermal stem and progenitor cells in murine epidermis are prone to the accumulation of cyclobutane pyrimidine dimers (CPDs) despite nucleotide excision repair (NER) proficiency suggests that human MSC will also accumulate more DNA damage in the form of CPDs during chronic UV irradiation [94, 95]. UVA irradiation has been shown to promote a greater number of oxidative DNA lesions in melanocytes than in keratinocytes, supporting the role that UVA may play in promoting DNA damage in cells of the melanocyte lineage including MSCs or melanocyte precursors [96].

Our extrafollicular dermal MSC model for the origin of melanoma predicts that any MSC residing in the dermis will accumulate DNA damage over the lifetime of an individual when protective and repair mechanisms are impaired due to the cumulative effects of UVA exposure from the sun or artificial sources such as tanning beds. Emerging evidence indicates that both quiescent (out of cell cycle and in a lower metabolic state) and active (in cell cycle and not able to retain DNA labels) stem cell subpopulations may coexist in several tissues in separate yet adjoining locations [61]. This may provide opportunities for MSCs to accumulate mutations when repair mechanisms are impaired. Severe sunburn in early childhood poses a significant risk for the development of melanoma well into adulthood [97, 98]. Damage to repair pathways will make MSCs more susceptible to subsequent radiation. When this occurs earlier in life, the MSCs will have a longer period of time to accumulate additional mutations [10]. Through the years, if they evade DNA repair and escape apoptosis due to defects in ROS defense mechanisms and damage response signaling pathways, MSCs will progressively accumulate genetic and epigenetic changes in their genome. We speculate that over time the dermal MSCs in different areas of the skin will accumulate a vast array of mutations due to exposure to different intensities of UVA from natural and artificial sources.

The importance of DNA repair in preventing the development of melanoma is illustrated in patients with xeroderma pigmentosum that have a defect in the nucleotide excision repair gene XPA and develop tumors with a high frequency on sun-exposed areas of the skin [99]. This suggests that DNA repair capacity plays an important role in preventing MSCs from accumulating UVA-induced DNA lesions that will tend to make any adult melanocyte developmentally derived from them to be more vulnerable to subsequent UVB-radiation when they migrate to the basal layer of the epidermis. Early onset of tumors and malignancy due to unrepaired DNA lesions, mutations or chromosomal modifications will then occur more often in sun-exposed areas of the skin.

MSCs ensure that adequate numbers of melanocytes are maintained so that keratinocytes receive enough melanin to protect the skin against the damaging rays of the sun. They regenerate melanocytes in response to damage and replace senescent melanocytes that no longer function. Age related loss of DNA damage repair pathways, through accumulated mutations from increased oxidative stress imposed by UV radiation, poses a significant threat to stem cell survival and function. Normal MSCs have strict control of gene expression and DNA replication whereas MSCs with loss of DNA repair may have altered patterns of proliferation, quiescence, and differentiation. Aging MSCs with loss of DNA repair may be more susceptible to malignant transformation upon subsequent exposure to intermittent UV [100]. Activation of DNA repair involves the participation of p53 [101]. Hair graying is a visible manifestation of aging MSCs and loss of self-renewal in the niche [102]. Thus, it is quite reasonable to suspect that the extrafollicular dermal MSC population in the skin is susceptible to UV-induced breakdown of DNA repair, bringing about increased genomic instability in these cells over time.

Mutated MSCs within the nerve sheath of peripheral nerves associated with skin may remain quiescent for years before they are called upon to replace severely damaged cutaneous melanocytes eliminated through apoptosis. How long the mutated MSCs remain quiescent will depend on the need of the skin to replace damaged cutaneous melanocytes. The extent of damage will depend on the nature and extent of UV radiation received by any given area of the skin over a certain period of time. For example, the intensity and frequency of both natural and artificial doses of UVA/UVB radiation experienced due to the lifestyles of any one individual, will certainly affect the lifespan of an epidermal melanocyte and thus how often the skin will need to signal a MSC to provide an replacement.

The duration as well as the precise stages of differentiation that a melanocyte precursor will go through as it migrates to a permanent residence on the basal layer of the epidermis is largely unknown. It may take several months or more to complete all four stages of melanocyte differentiation starting from the MSC (nerve-sheath precursor stage) and proceeding through the dermal and junctional migratory stages ending with the dendritic stage on the basal layer of the epidermis [24]. It is likely that transformation of an MSC itself or of a precursor during any of the stages leading up to a fully differentiated melanocyte will involve disruption of the normally tightly controlled process of self-renewal, stem cell expansion, differentiation and migration. Any factors which disrupt the normal physiology of the niche may trigger cascading effects and disrupt the normal homeostatic mechanisms regulating MSCs. Many of the signaling pathways regulating the regeneration of organ and tissues from stem cells overlap with those involved in pathways leading to carcinogenesis. This is consistent with the hypothesis that the earliest origins of melanoma begin in an extrafollicular dermal MSC when its molecular pathways regulating cell-cycle status are altered, leading to uncontrolled proliferation and abnormal differentiation [103].

Our model for the development of melanoma is based on the premise that a melanocyte precursor will be more vulnerable to UV-induced lesions than a fully differentiated melanocyte as it progresses through the different stages of maturation before reaching permanent residence on the basal layer of the epidermis. As a dermal MSC enters the melanocytic differentiation pathway and begins its migration to the epidermis to establish contact with the surrounding keratinocytes, it will gradually become more exposed to the less penetrating but more energetic UVB rays of sunlight. UVB radiation is directly absorbed by DNA and indirectly damages proteins and lipids by the formation of ROS. UVB-induced DNA modifications can lead to deleterious mutations, while oxidation of proteins and lipids may impair cell signaling pathways [85]. This continuum of possible target cells in the layers of the skin, created by the existence of melanocyte precursors at all stages along the differentiation pathway, may explain the different degrees of malignancy commonly seen in melanoma in different areas of the skin [104]. For example, aggressive forms of melanoma may reflect carcinogenic action in melanocyte precursors that were in the more primitive stages of the differentiation pathway [105]. Melanocyte precursors that have accumulated more mutations in critical repair and defense pathways but not yet transformed will have a higher probability of being transformed at later stages when they experience additional UV radiation from natural or artificial sources during their journey to the basal layer of the epidermis. Some melanocytes may reach the epidermis but will have compromised genomes, signaling proteins and antioxidant pathways making them more susceptible to transformation when exposed to high fluences of UVA/UVB irradiation.

Keratinocyte-derived growth factors and molecular crosstalk mediated by E-cadherin are likely to play a major role in regulating the activation and proliferation of abnormal melanocyte precursors immediately derived from extrafollicular dermal MSCs. Mutations in any of the genes regulating growth factors or crosstalk pathways may contribute to the transformation of a MSC at any stage of differentiation. The *β*-catenin gene is a likely candidate since it is generally involved in the self-renewal of stem cells and mutations of this gene have been found in patients with melanoma [106]. *β*-catenin dissociates from E-cadherin at the epithelial membrane and translocates to the nucleus where it activates transcription of WNT target genes [107]. Impaired *β*-catenin signaling is known to be linked to increased proliferation, abnormal differentiation and increased self-renewal of MSCs [108]. Increasing the frequency of MSC renewal will provide greater opportunities for accumulated DNA lesions to be converted to mutations in apoptotic and cell-cycle pathways, increasing the probability that either the MSC or its immediate melanocyte precursor will have a greater chance to transform into a melanoma stem cell.

### 2.5. Melanocyte Stem Cells in the Zebrafish

The zebrafish is becoming an ideal vertebrate system to study the interplay of those variables known to play a role in the development of melanoma. The melanocyte is common to both zebrafish and humans. A wealth of zebrafish pigmentation mutants are available that affect melanocyte specification, differentiation and function. Many of these genes have conserved roles in mammals and are nearly identical to humans [109]. Fish skin biology has major relevance to mammalian skin and offers a convenient animal model to gain molecular insights into regeneration and regulation of MSCs [110].

Our laboratory is currently utilizing the zebrafish model to investigate the effects of UVB/UVA radiation on MSCs. We are taking advantage of the recent discovery that the copper chelator, neocuproine (NCP), ablates adult melanocytes but not MSCs in the zebrafish. Following NCP washout, melanocytes regenerate from MSCs [38]. This is providing us with a very power technique to control the development of the entire melanocyte population in the zebrafish and synchronize their regeneration. Using this drug we can irradiate the entire stem cell population at one time. The drug-induced ablation of melanocytes can be done multiple times, permitting us to study how repeated rounds of UVA/UVB irradiation of MSCs affect the development of melanoma.

The regeneration of the adult zebrafish caudal fin is offering another opportunity to determine the effects of UVA/UVB irradiation on MSCs. Melanocytes in the regenerated fin arise from MSCs rather than from migration of previously differentiated melanocytes [111]. Furthermore, ontogenetic and regeneration melanocytes not only come from the same MSCs that colonize the fin, but also from the same MSCs responsible for growth and maintenance of the melanocyte pattern [112]. Studies of single progenitors or MSCs reveal no transfating or transdifferentiation between other lineages in the regenerating fin, showing that MSCs retain fate restriction when passed through the blastema [113]. This assures us that any damage to melanocyte pattern or proliferation that persists after additional amputations will be due to permanent genetic lesions in one or more MSCs. We stop the division and progression of melanocyte precursors from MSCs at different stages after amputation of the fin by using small-molecule inhibitors to achieve transient, reversible suppression of Wnt/*β*-catenin pathway [114, 115]. Thus, we have the capability to study the effects of UV irradiation on synchronized populations of melanocyte precursors at different stages of differentiation leading to a mature melanocyte. This is helping us to determine specific stages of increased sensitivity to UV and how increased UV at any one stage contributes to the development of melanoma.

## 3. Conclusion

Identifying the cell of origin for melanoma has a direct bearing on prognosis and chemoprevention strategies for melanoma. If melanoma has its origins in a MSC or in one of the melanocyte precursor stages that eventually leads to a fully differentiated melanocyte on the basal layer of the epidermis, it will be far better to target the pathways in these cells to keep self-renewal in check [116]. Identifying the molecular pathways and signaling molecules involved in MSC self-renewal and how these pathways are dysregulated by solar UV to produce a melanoma stem cell will be important for the development of more effective drugs for melanoma prevention and intervention [117]. If a damaged MSC or one of its precursors can be shown to be the cellular origin of melanoma, targeted identification and eradication by antibody-based therapies will be the best method to treat melanoma and a very effective way to prevent its recurrence [118]. The zebrafish model offers some powerful methods for investigating the role of MSCs in the development of melanoma.

## Figures and Tables

**Table 1:** Evidence for the presence of extrafollicular dermal MSCs in human skin and in association with peripheral nerves of other vertebrates.

Source	Origin	MSC marker	Marker type
Human	Dermis	kit (+), trp-1(−), bcl-2 (+)	Cytokine receptor, tyrosinase apoptosis regulator [6]
Human	Dermis	NGFRp75, Oct-4	Nerve growth factor [7–9]
Quail	Schwann cells	ETR-B	Endothelin receptor [29]
Chicken	Spinal ganglia	Melanin	Pigment [31]
Zebrafish	Dermis	trp-1 (−)	Tyrosinase [37–40]
Zebrafish	Nerve	foxd3 and sox10	Transcription factors [41]

**Table 2:** Genes involved in the development of melanocytes in humans, mice, and zebrafish.

Gene	Description	Humans	Mice	Zebrafish
MITF	Transcription factor	[62, 63]	[64]	[65]
PAX3	Transcription factor	[66]	[67]	[68]
SOX10	Transcription factor	[50, 52]	[69]	[70]
Wnt	Signaling protein	[71]	[72]	[73]
Notch	Membrane protein	[74, 75]	[76]	[77]
KIT	Cytokine receptor	[6, 78]	[79]	[5]
